# Association between older patients receiving geriatric co-management at the emergency department and acute hospital admissions compared to usual care: an observational, controlled study in the Netherlands

**DOI:** 10.1136/bmjopen-2025-101629

**Published:** 2026-04-16

**Authors:** Vera M Hogervorst, Marthe Ribbink, Rik van Eekelen, Bianca M Buurman, Annemarieke De Jonghe, Janet L Macneil Vroomen

**Affiliations:** 1 Department of Internal Medicine, Section of Geriatric Medicine, Amsterdam University Medical Centre, University of Amsterdam, Amsterdam, The Netherlands; 2 Department of Geriatric Medicine, Tergooi Medical Centre, Hilversum, The Netherlands; 3 Aging and Later Life, Amsterdam Public Health Research Institute, Amsterdam, The Netherlands; 4 Department of Epidemiology and Data Science, Amsterdam University Medical Centres, Amsterdam, The Netherlands

**Keywords:** Observational Study, GERIATRIC MEDICINE, Emergency Departments, Hospitalization, Health Services for the Aged, Frailty

## Abstract

**Abstract:**

**Objectives:**

The aim of this study is to determine if a geriatric co-management model, referred to as ‘The Geriatric Emergency Medicine (GEM)-team’ is associated with less admissions to hospital in older patients compared with the usual care without increasing the risk of mortality or 30-day emergency department (ED) readmissions.

**Design:**

This observational, controlled study used 18-month data prospectively collected from hospital records. Inverse probability weighting was used to account for baseline differences.

**Setting:**

An ED at a suburban Dutch general hospital, receiving approximately 10 000 patients aged 70 or older per year.

**Participants:**

All patients aged 70 or older were screened according to predefined criteria. When positively screened patients were presented at the ED on weekdays between 09:00–17:00, they received geriatric co-management. Outside these hours and when the capacity of the GEM team was reached, patients received care as usual.

**Interventions:**

Geriatric co-management at the ED involves a geriatric multidisciplinary team in collaboration with the primary ED physician who share management and responsibility for the provided medical treatment and nursing care starting directly at the primary assessment.

**Primary and secondary outcome measures:**

The primary outcome was hospital admission and secondary outcomes were the composite outcome of 30-day ED readmissions and mortality.

**Results:**

Patients receiving geriatric co-management (n=972) had lower odds for hospitalisation (OR: 0.77, 95% CI 0.65 to 0.91) compared with the control group (n=1355) while 30-day ED readmissions and mortality did not differ between groups (OR: 1.11, 95% CI 0.91 to 1.36).

**Conclusions:**

Geriatric co-management at the ED is associated with decreased hospital admissions while 30-day ED readmissions or mortality was not impacted. These preliminary results contribute to the evidence that geriatric co-management may be an effective intervention for older patients with frailty at the ED.

**STRENGTHS AND LIMITATIONS OF THIS STUDY**

- This study used real-world observational data from 2327 older patients, strengthening the generalisability of the data.
- Inverse probability weighting was used to address the limitations flowing from using observational data.
- A double robust approach and g-computation models were used for the sensitivity analyses.
- The novel add-on triage tool Geriatric Emergency Medicine-score was used, which is currently in the process of being validated.

## Introduction

 Emergency departments (EDs) are challenged by the multiple needs of the growing population of older patients with frailty,^1^ as the ED is originally designed to take care of single acute conditions such as heart attacks, fractures or infections.^2 3^ Therefore, older patients are frequently acutely admitted to hospital, which is associated with negative outcomes like mortality, long length of stay and discharges to other destinations than home.^4 5^ Assessing older patients living with frailty is complex because of non-specific and atypical presentation of illness, multiple comorbidities, polypharmacy, functional decline and altered homeostasis.^2 6^ Diagnostic challenges arise because of communication difficulties such as impaired memory functions, loss of hearing or speech and the need to communicate with informal caregivers.^7^ The complexity of care increases because of existential considerations related to altered perspectives on sickness and health in the light of the end of life nearing.^8 9^ Even older patients who have acquired a minor injury have a risk of unneeded, undesired and costly hospital admission because of all other patient-related factors.^10 11^

Interventions targeted at older patients at the ED have been introduced in different healthcare systems all over the world with variations within the exact models and resourcing.^12^,^15^ A holistic, multidomain assessment used by geriatric trained healthcare professionals called comprehensive geriatric assessment (CGA) is often included,^16^,^18^ sometimes combined with a discharge programme or a medication management programme. Evidence suggests that multistrategy interventions may be associated with reducing hospital admissions.^19^ Providing CGA at the ED is difficult because of the ED time pressures, costs and the increasing numbers of patients living with frailty.^20^ Examples of intervention models at the ED are separate geriatric EDs^21^ and secondary geriatric screening with prespecified screening lists by geriatric trained nurses.^22^ The evidence on how to provide a geriatric assessment at the ED is scarce and also costs are important to consider.^23 24^

Another known intervention model used in clinical settings as orthopaedics, oncology and cardiology is geriatric co-management, which uses the joint expertise of geriatric trained healthcare professionals and the primary physician.^25^ They share responsibility for treatment plans, discharge coordination and clinical outcomes. Geriatric co-management brings added resources and assistance, geriatric expertise and working relations with regional care partners.^26^ It is associated with reduced length of stay and complications throughout hospital admission.^25 27^ In the Netherlands, geriatric co-management, applied at the ED, is novel and has not been evaluated.

The aim of this study is to evaluate if a geriatric co-management model, referred to as ‘The Geriatric Emergency Medicine (GEM)-team’ is associated with less acute admissions to hospital in older patients compared with usual care without increasing the risk of mortality or 30-day ED readmissions.

## Methods

### Study design

This observational, controlled study evaluated patients that were presented to the ED and co-managed by the GEM team compared with a control group who received usual care for an 18-month period. Anonymised data retrieved from the electronic health records (EHRs) was used for this study. The Strengthening the Reporting of Observational Studies in Epidemiology^28^ and the Template for Intervention Description and Replication (TIDieR) guidelines (online supplemental file 1) were used for reporting.

### Setting

In May 2020, the GEM team was implemented between 09:00–17:00 on weekdays, on a 20-bed ED at one of the two locations of a general teaching hospital located in a suburban area in the Netherlands. This ED served patients with surgical, orthopaedic or internal medicine problems. Within the catchment area of the hospital, no other hospital exists. During the study period, no short stay area was available for ED patients. In 2018 a regional covenant between the hospital and regional intermediate care facilities was established. The covenant describes the agreements and logistics regarding transfer of ED patients to an intermediate care facility at all hours.

### Participants and intervention

All patients aged 70 or older who visited the ED during the 18 months’ time window were triaged at presentation by the ED nurse. The ED nurse then applied the GEM score to decide if geriatric co-management was indicated. The GEM score quickly screens for the presence of cognitive problems, delirium, fall/collapse-related presentation or anticipated discharge problems. The last item of the GEM score was the opinion of the ED nurse whether the patient would benefit from geriatric co-management. Cognitive problems were screened for by reviewing medical notes or hand-over for a recorded cognitive disorder diagnosis or by verbal confirmation of next-of-kin. The presence of delirium was assessed by an ED nurse and, if needed, a confirmation of the next-of-kin about a recent change in behaviour of the patient. The GEM score has a binary outcome, yes or no. If one or more items of the GEM score were positively confirmed, the GEM team started their geriatric co-management trajectory. While the GEM score is not a validated tool, it is based on validated scores as the Veiligheids Management System score, Acuut Presenterende Oudere Patient score and Identification of Seniors At Risk-Hospitalised Patients^29^ and a pragmatic expert opinion of the geriatric and ED team at the hospital. The inclusion of positively GEM score screened patients in the GEM cohort was mainly time-dependent but also influenced by the availability of the advanced nurse practitioner (ANP) or medical doctor (MD) of the GEM team. When too many GEM patients were presented at once at the ED, inclusion in the GEM cohort was left to the discretion of the GEM ANP/MD. Patients with high risk for unnecessary hospital admission and/or considered highly frail, determined by the Clinical Frailty Score,^30^ were then favoured.

**Figure 1:**
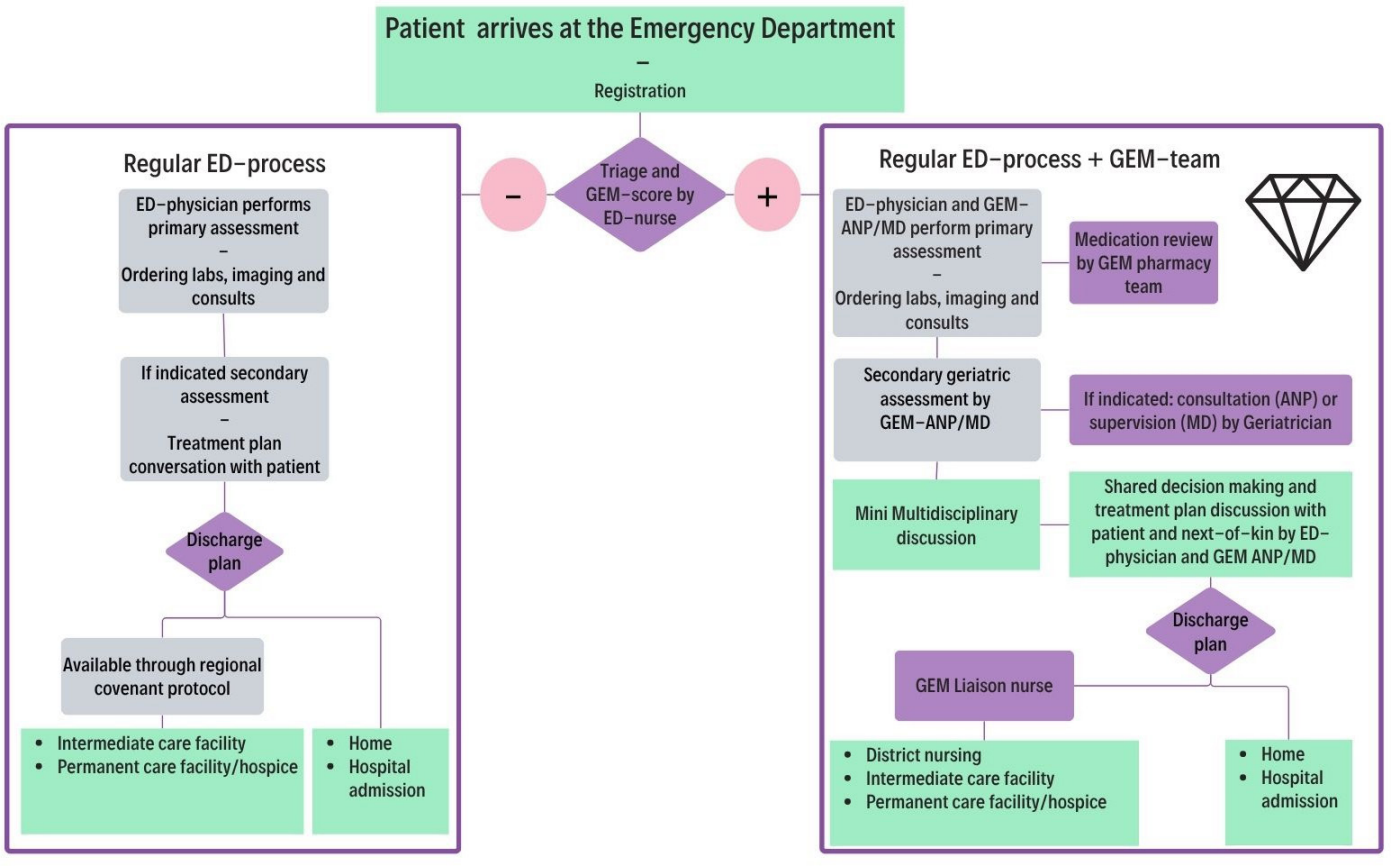
The Geriatric Emergency Medicine (GEM)-team trajectory at the Emergency Department. ANP, advanced nurse practitioner; ED, emergency department; MD, medical doctor.

The GEM team is a collaboration of an ANP or MD trained in GEM; ED nurses; a geriatrician; a pharmacy team and liaison nurses. In the Netherlands, ANPs are senior nurses with a master’s degree and 2 years of medical training into a prespecified medical expertise area. They are legally authorised to autonomously provide integrated medical and nursing assessments.^31^ The MD is supervised by the geriatrician and was provided with on-site training regarding assessing older patients at the ED at our teaching hospital. The ANP liaises with the geriatrician when the assessment of the patient exceeds their medical expertise. Both the ANP and the MD provide an integrated medical and nursing geriatric assessment based on the CGA framework adapted to the urgent care setting^18^ in co-management with the primary ED physician. The GEM team is based at the ED and was assigned to perform CGA within the time window the primary ED physician needed to assess the patient. The GEM team complements the primary ED physician by simultaneously working together starting at the primary assessment. They decided together which laboratory tests, imaging and consults were needed. The GEM team ANP/MD considered treatment preferences and advance care directives of the patient. Special attention was given to non-specific problems and atypical presentation of illness, comorbidities, functional decline and altered homeostasis, as these are common pitfalls known in the assessment of older patients. Polypharmacy and drug-related ED presentations were assessed together with the pharmacy team.^32^ The GEM team ANP/MD collaborated with the ED nurses regarding secondary geriatric nursing screens, investigating the social network of the patient, indicating preventive measures and providing a geriatric-friendly stay at the ED. When all tests and consultations have taken place, a quick mini multidisciplinary discussion is being held which further enrols into talking with the patient and next of kin regarding treatment options, advanced care directives and the location of the treatment and care (hospital, permanent/intermediate care facility or at home) using shared decision making. When treatment and care at a permanent or intermediate care facility was chosen or district nursing at home, the hospital-based liaison nurses organised the transfer in collaboration with the regional healthcare providers. When hospital treatment is not indicated or desired, preference is to transfer the patient home or to a permanent or intermediate care facility. At discharge, the GEM team ANP/MD provided the patient and next of kin with discharge information and follow-up appointments. A written hand-over and/or telephone call with the regional healthcare providers is conducted at discharge of the ED. At admission to the hospital, treatment instructions were written at the electronic health records (EHRs) and verbally handed over. If indicated, a geriatric co-management trajectory throughout hospital admission was initiated (figure 1).

**Figure 2:**
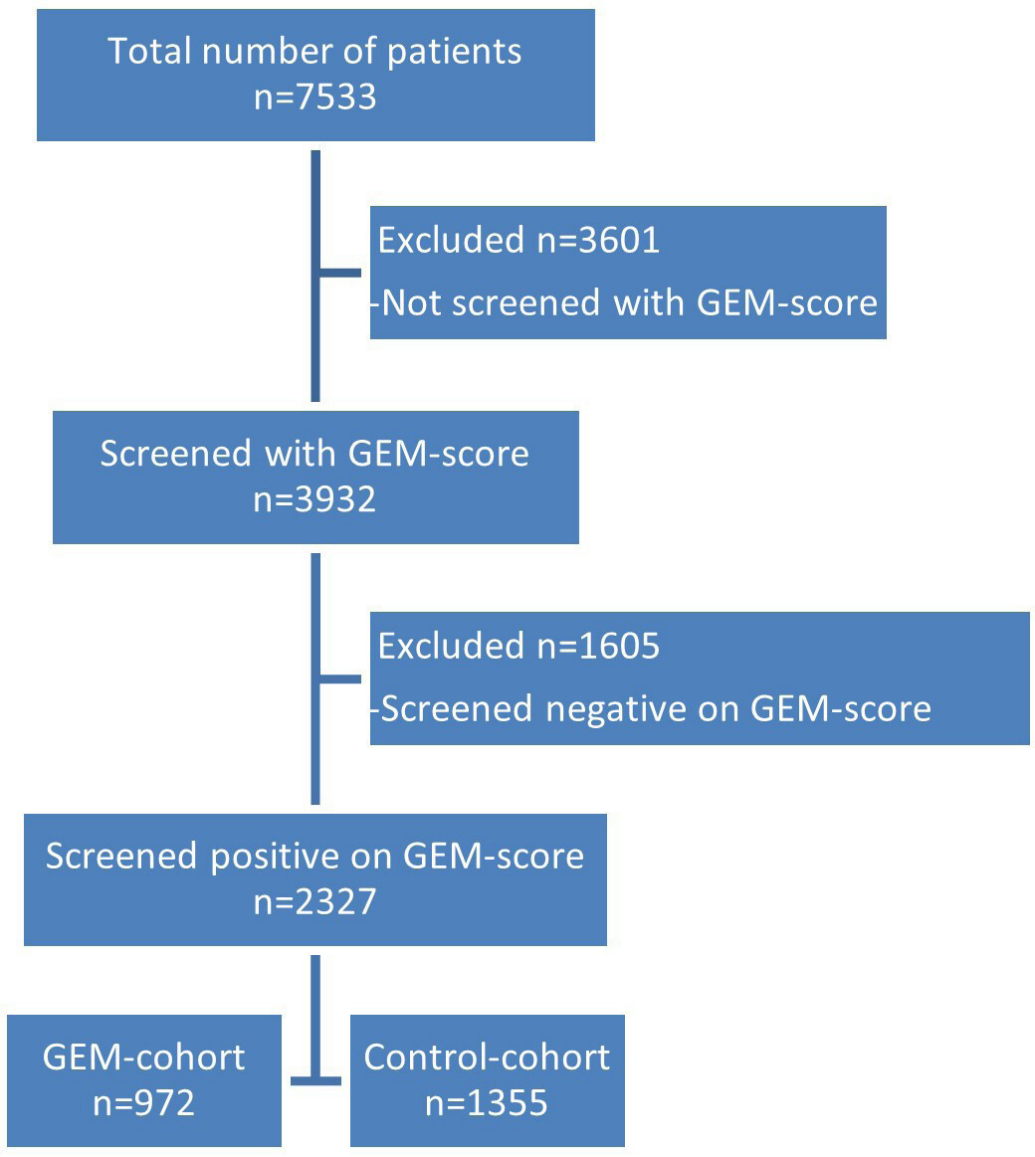
Enrolment of patients, 70 years and older, presented at the emergency department. GEM, Geriatric Emergency Medicine.

For this study, two cohorts were composed of all consecutively included patients aged 70 or older presented at the ED between May 2020 and November 2021. All patients not screened with the GEM score or screened with the GEM score but scored negatively were excluded. Patients screened positively at the GEM score and co-managed by the GEM team formed the GEM cohort, and patients screened positively but not seen by the GEM team formed the control cohort (figure 2). This cohort received care as usual, including the possibilities created with the regional covenant.

### Data Collection

18 months of data were prospectively collected at the EHR of the patient. As part of the CGA, the Clinical Frailty Scale was assessed^33^ and discharge settings and/or destinations were noted. Data was anonymously retrieved from the EHR and municipal records with the help of administrative staff. Baseline data included gender, age at presentation, date and time of ED presentation, triage urgency and medical specialty of the primary ED physician. Collected outcome data included date of death, admission to hospital and discharge dates and 30-day ED-readmission dates. All data was uploaded in SPSS V.28 and R V.4.0 for analyses. Missing data regarding baseline characteristics was recorded in table 1 and accepted. Outcome data was available and calculated for all patients. Data checks were performed. Acute admission to hospital was defined as: being transferred from the ED to a clinical ward or theatre at the hospital and having a hospital stay larger than 24 hours (including the hours at the ED). ED admissions usually take around 2–3 hours and have never exceeded 24 hours. All members of the GEM team and some investigators based at the general hospital had access to parts of the EHR. To minimise chances of bias, it was chosen to perform the analyses on the anonymised data files in collaboration with external investigators based at the academic hospital.

**Table 1:** Baseline characteristics of all patients, unweighted results

Patient characteristics	GEM cohort n=972	Control cohort n=1355	P value	Standardised mean difference
Age in years, mean (SD)	83.3 (7.0)	82.7 (7.0)	0.031	0.091
Gender (female), n (%)	594 (61.1)	818 (60.4)	0.750	0.015
Triage priority level (MTS), n (%)			<0.001	0.180
Immediate/very urgent	91 (9.4)	186 (13.7)		
Urgent	351 (36.1)	537 (39.6)		
Standard/non-urgent	530 (54.5)	632 (46.6)		
Presented at the ED between 09:00–17:00 on weekdays, n (%)	909 (93.5)	564 (41.6)	<0.001	1.332
Cognitive impairment, n (%)	309 (31.8)	404 (29.8)	0.330	0.043
(High risk of) delirium, n (%)	242 (24.9)	359 (26.5)	0.412	0.037
Presented at ED with fall/collapse, n (%)	611 (62.9)	715 (52.8)	<0.001	0.205
Expected discharge problems*, n (%)	537 (55.2)	787 (58.1)	0.187	0.057
ED-nurse indicates geriatric co-management at the ED, n (%)	638 (65.6)	896 (66.1)	0.841	0.010
Treating physician surgical (vs/non-surgical), n (%) Missing data, n	698 (71.8) 35	802 (59.2) 53	<0.001	0.268

P value: comparison of variable between GEM cohort and Control cohort, using t-test or χ² test, statistical significance with alpha=0.05.

* At presentation at the ED, the ED nurse estimates whether discharge problems, related to insufficient care available at the living situation of the patient, are to be expected.

ED, emergency department; GEM, Geriatric Emergency Medicine; MTS, Manchester Triage System.

### Outcomes

The primary outcome of the analyses was the odds of being acutely admitted to the hospital. Secondary outcomes were a composite of 30-day ED readmission and mortality to adjust for competing risks, 90-day mortality and hospital length of stay.

### Analysis

This observational pragmatic study used all available data. Baseline categorical data are presented as frequencies and percentages, continuous variables with mean and SD. The standardised mean differences for characteristics between intervention groups were estimated to help show the order of magnitude. To weight the samples in each group so that the distributions of preintervention characteristics were similar between the two groups,^34^ inverse probability weighting (IPW) was used using the ipw package in R.^35^ The confounders included were age, gender, triage priority level, primary ED-physician, presence of cognitive problems, delirium, fall/collapse-related presentation, anticipated discharge problems and presence of an indication for geriatric co-management by the ED nurse. Age was included as non-linear using restricted cubic splines with five knots. The weights were standardised and truncated at the 1st and 99th percentile. The standardised mean differences between baseline characteristics and intervention groups were reassessed after weighting. Crude (ie, unadjusted and unweighted) estimates of the effect of intervention were estimated and then adjusted after IPW by using weighted logistic regression on binary outcomes. For hospital length of stay, the regular and then the weighted Mann-Whitney U test was used.

### Sensitivity analysis

Two sensitivity analyses were incorporated: first, the weighted outcome model (ie, logistic regression for the primary outcome admission) was also adjusted for the propensity score, which is a simple form of a doubly robust approach. Second, g-computation^36^ was conducted. A logistic regression model with all the same confounders that were used in the IPW approach was fitted instead of estimating a propensity score model. Every individual participant’s probability for hospital admission based on their covariates was estimated, both with and without the intervention. The probabilities were averaged separately under the two conditions and used to estimate the (marginal) OR. A non-parametric bootstrap was used with 1000 repetitions to estimate the SE and obtain 95% CIs.

### Patient and public involvement

The implementation process of the GEM team contained analyses of patient journeys and conducting 18 patient and next-of-kin interviews pre-implementation and post implementation. These were not analysed in this article. The local patient advisory board was consulted. The outcomes were discussed by the GEM team and the hospital management. Action items were implemented throughout implementation.

## Results

Approximately 7533 patients aged 70 or older presented at the ED during the study time. A total of 3932 (52.2%) patients were screened with the GEM score and 2327 scored positively. 3601 patients were not screened with the GEM score and excluded from this study (figure 2).

### Descriptive characteristics

Table 1 describes the baseline characteristics of the GEM cohort and control cohort, before weighting. The GEM cohort patients are older and more likely to present with a fall/collapse at the ED. The triage priority level according to the Manchester Triage System was overall lower for the GEM cohort. The GEM cohort was largely seen during weekdays between 0900–17:00 and the primary ED physician was more often a surgical doctor. The average Clinical Frailty Score for the GEM cohort was five. In online supplemental file 2, the baseline characteristics before and after weighting are provided. All standardised mean differences were below 0.10. The weights were relatively stable with an average of 1.00 and a range of 0.78–1.47.

### Primary outcome

After weighting, geriatric co-management at the ED for older patients was associated with decreased odds (OR: 0.77 (95% CI 0.65 to 0.91)) of hospital admission compared with the control cohort.

### Secondary outcomes

No evidence was found to suggest that geriatric co-management at the ED could be associated with the composite outcome of 30-day ED readmissions and the competing risk of 30-day mortality: the weighted OR was 1.11 (95% CI 0.91 to −1.36). The remaining secondary outcomes are listed in table 2 as well as all crude estimates.

**Table 2:** Outcomes of patients co-managed at the ED by the GEM team compared with the control cohort

Outcome	GEM cohort n, (%)	Control cohort n, (%)	Crude estimate, OR (95% CI)	IPW estimate, OR (95% CI)
Admission to hospital*	550 (56.6)	898 (66.3)	0.66 (0.56 to 0.79)	0.77 (0.65 to 0.91)
Composite of 30-day ED readmission or death	199 (20.5)	275 (20.3)	1.01 (0.82 to 1.24)	1.11 (0.91 to 1.36)
30-day ED readmission	107 (11.0)	158 (11.7)	0.94 (0.72 to 1.21)	1.00 (0.77 to 1.29)
30-day all-cause mortality	100 (10.3)	133 (9.8)	1.05 (0.80 to 1.38)	1.18 (0.89 to 1.55)
90-day all-cause mortality	156 (16.0)	199 (14.7)	1.11 (0.88 to 1.39)	1.25 (0.99 to 1.56)
Length of stay during hospital admission Mean difference, Mann-Whitney U	7.07 (9.8)	6.93 (8.9)	−0.6, p<0.001	−0.3, p=0.021

* Primary outcome.

ED, emergency department; GEM, Geriatric Emergency Medicine; IPW, inverse probability weighting.

### Sensitivity analyses

Results for the doubly robust approach (adjustment for propensity score after weighting) and results for g-computation were similar to the main analysis. For doubly robust, the estimated OR was 0.76 (95% CI 0.64 to 0.91) and for g-computation this was 0.77 (95% CI 0.66 to 0.91).

### Outcomes specifically for the GEM cohort

**Figure 3:**
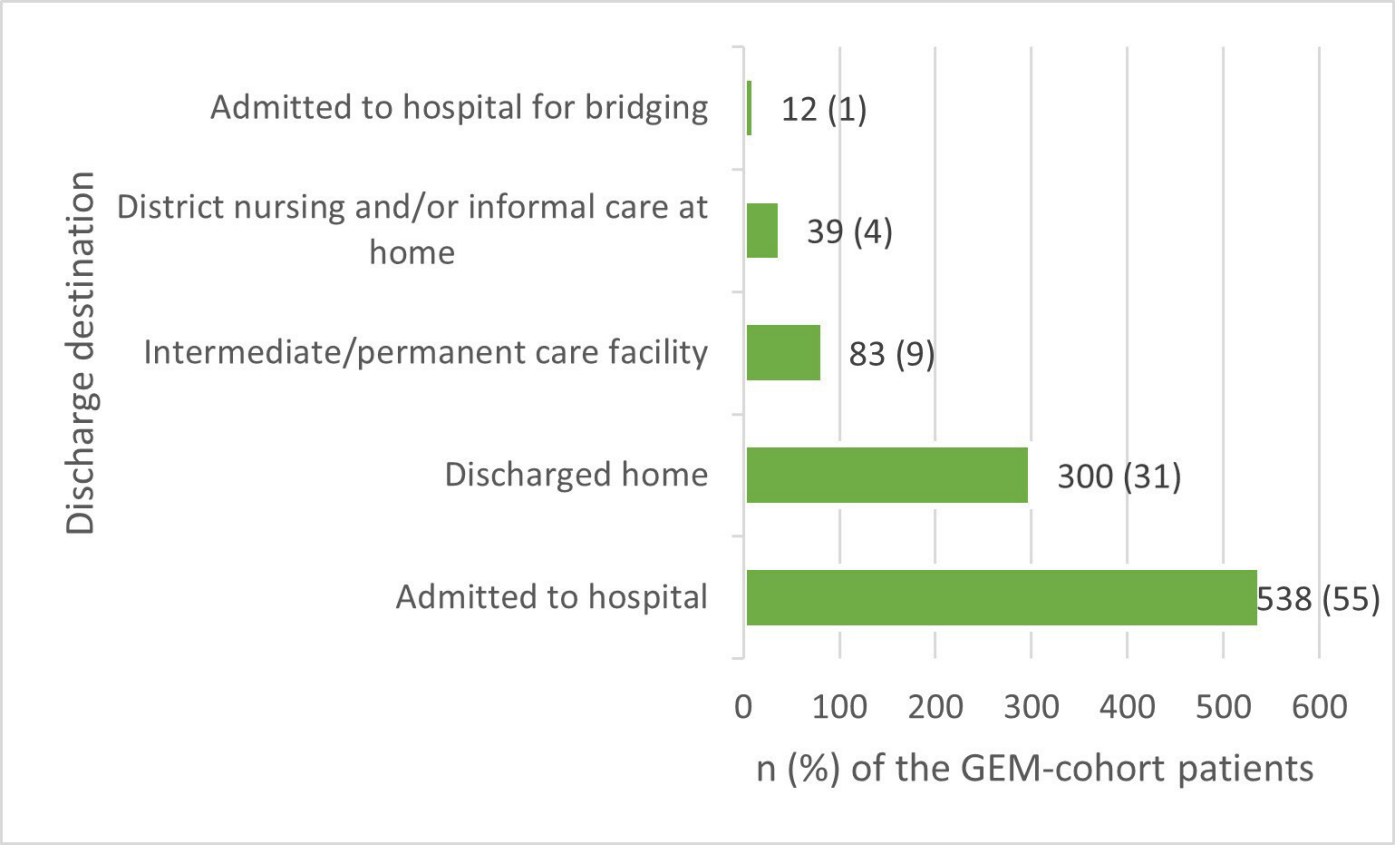
Discharge destinations GEM cohort: n (%). GEM, Geriatric Emergency Medicine.

The discharge destinations of the GEM team cohort are summarised in figure 3, to illustrate how many actual transfers to an intermediate or permanent care facility or home did take place, resulting in the lower odds for hospital admission. For the control cohort, the discharge destinations are not known other than admitted to the hospital or not.

## Discussion

This study found that geriatric co-management at the ED is associated with decreased hospital admissions, without having a negative effect on the 30-day ED readmission rates, mortality within 30 or 90 days. These preliminary findings show that the concept of geriatric co-management can effectively be transferred to the ED and is associated with beneficial outcomes as lower admission to hospital rates without putting the patient at risk for relevant negative outcomes. Examples of intervention components are recognition of non-specific problems and atypical presentation of illness, attention for relevant comorbidities, robust medication reviews,^32^ consistent attention for advance care directives and patient preferences, and ongoing working relations with the regional care partners.^11 37 38^

### Strengths and limitations

The strength of this observational study was the inclusion of all older patients presenting at the ED, which represented a real-world population and increased generalisability. This contributes to the feasibility of implementing geriatric co-management at the ED. By using real-world data, this study bridges the gap between evidence and implementation.

A limitation of this study was the study design as the intervention and data inclusion coincided with the first SARS-CoV-2 epidemic period, possibly affecting outcomes and making a preferred before and after design impossible. However, a control cohort out of the same time window was created.

To address the limitations of observational data, IPW was used, with a double robust approach and g-computation models for the sensitivity analysis.

Another limitation is the GEM score screening. The novel add-on triage tool, the GEM score, is currently undergoing validation. Although the ED nurses were instructed to apply the GEM score to all aged 70 or older, only 52% were screened. Incorporating the GEM score into regular work processes proved difficult, especially during out-of-office hours.

One more limitation is timing and selection bias. The GEM team was available on weekdays between 09:00–17:00. This created a substantial baseline difference: 94% of the patients in the GEM cohort were seen between these hours versus 42% of the control cohort. This is a large imbalance which resulted in broad, skewed distributions and unstable weights unsuitable for analyses. To address this imbalance, propensity score methods were implemented to adjust for the effects of differential presentation times, effectively controlling for this baseline difference. Approximately 58% of the control cohort that was presented outside working hours of the GEM team possibly had a higher risk of hospital admission related to being sicker and in need of immediate ED presentation. A propensity score was created, including triage priority level, presence of cognitive problems, delirium, falls and anticipated discharge problems. The triage priority level represents an assessment of the severity of disease and an interpretation of the vital signs.

Additionally, the control-cohort patients had access to the regional covenant protocol, which made it possible to transfer ED patients to an intermediate care facility at all hours. This covenant addresses the possible effect of less availability of resources out of office hours. It was not chosen to just compare those presented under office hours, as those who were not seen by the GEM team during office hours were considered less frail and/or having a reduced risk of unnecessary hospital admission, resulting in a bias in favour of this potential control group. However, some confounding related to the hours of presentation cannot be excluded. Therefore, this study limits itself to assessing an association and not a causal relation. Because of the use of real-world data, the gap between evidence and implementation is bridged. However, this study is limited in assessing the GEM team’s internal validity as causal effects cannot be established with this study design.^39^

The anonymised data available to this study was limited and much depended on regular hospital systems and possibilities within privacy regulations. Due to a change in hospital systems, it was not possible to analyse length of stay at the ED. The strength of the collaboration of the GEM team and the regional network is of influence on the generalisability of these study results.

These findings are similar to an Australian study where a multidisciplinary team performed targeted geriatric assessment and focused on streamlining of care at the ED.^15^ This intervention design has similarities to our geriatric co-management model; however, in the Australian intervention, primary assessment was done by an ED physician alone and selection of the patients was left to the discretion of the multidisciplinary team. No pharmacy team was involved. Geriatric co-management is expected to bring shared responsibility for treatment plans, added resources and assistance, geriatric expertise and working relations with regional care partners.^26^ By adding this at the start of the primary assessment for a selected group of patients, it is thought that consistent care for a large group of older patients can be delivered. Leaving the responsibility of an important part of the trajectory at the ED with the ED physician might create dilemmas in prioritising important but not life-threatening care. In the USA, where numbers of dedicated geriatric EDs based on geriatric ED guidelines are increasing,^40^ similar outcomes to our study are found. However, in our intervention, it was chosen to create a team that relies on working relations with regional care partners. The Dutch healthcare system generally has a stronger primary care coordination system compared with the USA,^41^ and Dutch governmental healthcare bodies stimulate regional collaboration as a strategy to keep the healthcare system accessible.

In contrast to the outcomes of a systematic review suggesting that multistrategy interventions and a large follow-up may be associated with a reduction of hospital admission and 30-day ED readmissions,^19^ geriatric co-management did not result in lower risk for 30-day ED readmissions. This might be related to the short time of the intervention only during the ED presentation and no long-term follow-up.

### Recommendations

Geriatric co-management was already seen as a promising approach to hospital care.^27^ These preliminary study results could imply that geriatric co-management at the ED might be of interest too. It contributes to an integrated healthcare system approach, which will be needed to keep our healthcare system accessible during the demographic changes heading our way in the coming years.^42^ In the Netherlands, it is of high priority to only use hospital beds for those who need and desire treatment. As the GEM team helps to select the right patients for transmission to home or an intermediate or long-term care facility, it is possible to deliver more care without the need for more expensive clinical beds. Presentation to the ED by patients living with frailty cannot always be avoided as quick and urgent clinical assessments might be indicated, but admission to hospital without a need or desire for treatment can be prevented.

Future studies would be to evaluate the GEM team and its association with quality of life, patient experiences and patient-related outcomes. Multicentre studies with clustered randomised controlled trial cross-over designs could be considered. The GEM score needs to be evaluated and perspectives on the implementation process might be of benefit to generalise these results. It would be of interest to evaluate the effectiveness of geriatric co-management at the ED in other healthcare systems.

## Conclusion

Geriatric co-management at the ED is associated with decreased hospital admissions while 30-day ED readmissions or mortality was not impacted. These preliminary results contribute to the evidence that geriatric co-management may be an effective intervention for older patients with frailty at the ED.

## Supplementary material

10.1136/bmjopen-2025-101629 online supplemental file 1

10.1136/bmjopen-2025-101629 online supplemental file 2

## Data Availability

Data are available upon reasonable request.
