# Supplementary material for: Association between older patients receiving geriatric co-management at the emergency department and acute hospital admissions compared to usual care: an observational, controlled study in the Netherlands
Source: BMJ Open. 2026 Apr 16;16(4):e101629. doi: 10.1136/bmjopen-2025-101629 (PMC13110556; doi:10.1136/bmjopen-2025-101629)
Supplement: online supplemental file 2 [file bmjopen-16-4-s002.pdf]

ADDITIONAL FILE 2 BASELINE CHARACTERISTICS OF ALL PATIENTS, UNWEIGHTED AND WEIGHTED RESULTS

	Before propensity score weighting			After propensity score weighting		
Patient characteristics	GEM-cohort n=972	Control-cohort n=1355	Standardized mean difference	GEM-cohort n=971	Control-cohort n=1355	Standardized mean difference
Age in years, mean (SD) Unweighted	83.3 (7.0)	82.7 (7.0)	0.091	83.0 (7.0)	82.9 (7.0)	0.003
Gender (female), n (%)	594 (61.1)	818 (60.4)	0.015	589 (60.7)	823 (60.7)	0.001
Triage priority level (MTS), n (%)			0.180			0.002
• Immediate/ Very Urgent	91 (9.4)	186 (13.7)		117 (12.2)	163 (12.0)	
• Urgent	351 (36.1)	537 (39.6)		369 (38.0)	516 (38.1)	
• Standard/ Non-Urgent	530 (54.5)	632 (46.6)		485 (49.9)	676 (49.9)	
Presented at the ED between 9AM and 5PM on weekdays, n (%)	909 (93.5)	564 (41.6)	1.332	907 (93.4)	559 (41.2)	1.338
Cognitive impairment, n (%)	309 (31.8)	404 (29.8)	0.043	297 (30.6)	415 (30.6)	0.001
(High risk of) Delirium, n (%)	242 (24.9)	359 (26.5)	0.037	260 (26.7)	354 (26.1)	0.013

Presented at ED with Fall/collapse, n (%)	611 (62.9)	715 (52.8)	0.205	555 (57.1)	772 (57.0)	0.002
Expected discharge problems, n (%)	537 (55.2)	568 (41.9)	0.057	552 (56.8)	772 (57.0)	0.004
ED-nurse indicates Geriatric co-management at the ED, n (%)	638 (65.6)	896 (66.1)	0.010	639 (65.8)	892 (65.9)	0.001
Treating physician surgical (vs. non-surgical), n (%)	698 (71.8)	802 (59.2)	0.268	626 (64.4)	872 (64.4)	0.001
• Missing data, n	35	53				
