# Supplementary material for: Association between older patients receiving geriatric co-management at the emergency department and acute hospital admissions compared to usual care: an observational, controlled study in the Netherlands
Source: BMJ Open. 2026 Apr 16;16(4):e101629. doi: 10.1136/bmjopen-2025-101629 (PMC13110556; doi:10.1136/bmjopen-2025-101629)
Supplement: Supplementary file 3 [file bmjopen-2025-101629.reviewer_comments.pdf]

PEER REVIEW HISTORY

BMJ Open publishes all reviews undertaken for accepted manuscripts. Reviewers are asked to complete a checklist review form and are provided with free text boxes to elaborate on their assessment. These free text comments are reproduced below.

ARTICLE DETAILS

Title (Provisional)

Association between older patients receiving geriatric co-management at the emergency department and acute hospital admissions compared to usual care: an observational, controlled study in the Netherlands.

Authors

Hogervorst, Vera; Ribbink, Marthe; van Eekelen, Rik; Buurman, Bianca; De Jonghe, Annemarieke; Macneil-Vroomen , Janet L.

VERSION 1 - REVIEW

**Reviewer**	**1**
**Name**	**Tekin, Erdal**
**Affiliation**	**Ataturk University, Emergency Medicine**
**Date**	**16-May-2025**
**COI**	**None**

I read your observational and controlled study conducted on a geriatric patient population in the emergency department with great interest. The elderly patient group is a special population and their emergency approaches should be different. In this respect, I think it is an interesting study. I have a few suggestions for your study. These are;

The introduction section can be shortened and written more concisely and neatly.

Can more information be provided about the clinical accuracy, specificity and sensitivity of the GEM score used in the study?

**Reviewer**	**2**
**Name**	**Pines, Jesse**
**Affiliation**	**George Washington University**
**Date**	**06-Nov-2025**
**COI**	**None**

Overall, this is a well-written and clinically relevant observational study examining the link between geriatric co-management (the GEM-team model) in the emergency department and hospital admissions among older adults.

The authors use inverse probability weighting based on a propensity score to adjust for differences between groups. Yet, because assignment to the intervention was largely determined by time of day and team availability, residual confounding remains a major concern given time of day can influence clinical presentations, severity of illness, ED resources, and ultimately hospital admission which is the primary outcome.

The propensity model does not fully account for this structural imbalance between patients seen during GEM operating hours and those receiving usual care. Consequently, "apples-to-apples" comparability is limited. However, with stronger adjustment for time-related and staffing factors, inclusion of balance diagnostics, and additional sensitivity analyses, I might be more convinced of the results.

Major recommendations:

Regarding the presentation (overall it is nicely written):

1. The introduction is very long. The intervention should be described primarily in the methods. Consider moving the details of the intervention to the methods.

2. Regarding the discussion, it should be somewhat reframed to emphasize association rater than causation.

Regarding the primary analysis (I have concerns regarding the confounding by time of presentation):

1. Add time-of-presentation (hour, weekday/weekend) as covariate(s) in the propensity model.

2. Provide covariate-balance plots (standardized mean differences) before and after weighting.

3. Conduct the following sensitivity analyses:

o Restrict analysis to patients presenting within GEM operating hours ("within-hours subset") to test robustness

o Use doubly robust models (IPW + covariate adjustment)

o Explore alternative specifications (e.g., matching with calipers, entropy balancing).

4. Consider propensity-score matching rather than weighting, reporting matched-sample characteristics and effective sample size.

5. Report the distribution of propensity scores to confirm overlap.

Minor recommendations

1. Add the number of excluded unscreened patients.

2. Include figure or table showing standardized mean differences before/after weighting.

VERSION 1 - AUTHOR RESPONSE

Response to reviewer's comments:

Reviewer's comments	Response	Location and citation of edits
The introduction section can be shortened and written more concisely and neatly.	We thank the reviewer for this comment. Alterations have been made to this paragraph and the description of the intervention has been moved to the methodology section	Introduction
Can more information be provided about the clinical accuracy, specificity and sensitivity of the GEM score used in the study?	We thank the reviewer for this important question. Both the Geriatric Emergency Medicine (GEM)-team and the GEM-score are novel interventions. This paper represents the first preliminary evaluation of the GEM-team intervention, and we acknowledge that detailed psychometric properties of the GEM score are not yet available. A formal validation study of the GEM score is currently underway to assess its clinical accuracy, sensitivity, and specificity. We expect to publish these findings in the near future.	-
The authors use inverse probability weighting based on a propensity score to adjust for differences between groups. Yet, because assignment to the intervention was largely determined by time of day and team availability, residual confounding remains a major concern given time of day can influence clinical presentations, severity of illness, ED resources, and ultimately hospital	We thank the reviewer for this comment. We agree that this is one of the major concerns related to this paper, which we tried to address carefully. We do recognise that this difference in groups is a limitation, related to the set-up of this semi-experimental study using observational data out of electronic patient files. We have used IPW to reduce bias as much as possible. Originally, we included time of presentation in our models, but this resulted in unstable and skewed weights. This is explained by the fact that the inclusion in the GEM-team intervention cohort was only possible if the patient presented during working hours of the GEM-team.<br>We then opted not to adjust for time of presentation but instead for prognostic factors that might differ due to presenting hours:<br><br>- Severity of disease was adjusted for using triage priority level representing the severity of disease and an impression of the vital signs at presentation at the emergency department (ED). Also the factors presence of cognitive problems,	-Discussion (strength and limitations)<br>-Methods (analyses)<br>-Results<br>-Additional file 2

<table>
<tr>
<td>admission which is the primary outcome.
The propensity model does not fully account for this structural imbalance between patients seen during GEM operating hours and those receiving usual care. Consequently, “apples-to-apples” comparability is limited. However, with stronger adjustment for time-related and staffing factors, inclusion of balance diagnostics, and additional sensitivity analyses, I might be more convinced of the results.</td>
<td>delirium, falls and anticipated discharge problems were used.

- ED resources: One of the problems the GEM-team addresses is indeed the provision of resources to make desirable outcomes as lower admission rates to hospital possible. For the control cohort an alternative was present by a regional covenant making it possible to transfer patients to care facilities at all hours. Please note that the control-cohort contains many patients (42%) that presented at the ED during office hours at times the GEM-team was unable to attend all GEM-score positive patients. When this situation occurred, the GEM-team ANP/MD quickly analysed which patients had a low Clinical Frailty Score – representing that they were deemed less frail- and had a low chance of undesired hospital admission. Based on those observations it was decided which patients should be seen by the GEM-team and which not. Patients with a higher Clinical Frailty Score and higher change of undesired hospital admission were preferred. The patients who could not be seen by the GEM-team were then added to the control-cohort. Doing this, we were aware that these control-cohort patients would, on average, be at a lower risk of hospital admission. This means that our results are conservative instead of optimistic.

Furthermore, as a response to your comments, we have asked an extra statistician (RvE), specialised in statistical methodology related to semi-experimental studies, to join our authors team and have recalculated all our analyses according to current and updated scientific insights regarding the use of propensity scores and inverse probability weighting. Hence, an insignificant difference in baseline diagnostics (see table 1) has occurred, but this did not impact the effects and statistical significance of our outcomes. Balance diagnostics and sensitivity analyses have been added now with the update of this paper. The average of the standardized weights was 1.00 with a range of 0.78 to 1.47, indicating relatively stable weights.

The residual bias is a limitation of this study and we have described this in our discussion section as a limitation.</td>
<td></td>
</tr>
<tr>
<td>The introduction is very long. The</td>
<td>Thank you for this comment, we have rewritten parts of the introduction to be more concisely and have indeed</td>
<td>Introduction</td>
</tr>
</table>

intervention should be described primarily in the methods. Consider moving the details of the intervention to the methods.	removed the description of the intervention from the introduction.	
Regarding the discussion, it should be somewhat reframed to emphasize association rather than causation.	We thank the reviewer for this important comment. We agree that the observational study design limits our ability to infer causality, and it was never our intention to suggest causal relationships. We have revised the discussion section to consistently emphasize associations rather than causal effects. Specifically, we have modified language throughout to use terms such as "associated with," "related to," and "linked to" rather than implying direct causal relationships.	Discussion
Add time-of-presentation (hour, weekday/weekend) as covariate(s) in the propensity model.	We thank the reviewer for this comment. We refer to the earlier comment regarding that the propensity model does not fully account for this structural imbalance between patients seen during GEM operating hours and those receiving usual care. We have revised the discussion section to provide more transparency regarding our choices for the analyses and the limitations of the study.	Discussion (strengths and limitations)
Provide covariate-balance plots (standardized mean differences) before and after weighting.	With the help of our new team member, a statistician specialised in this methodology, we have redone all our analyses based on the current scientific insight on this methodology. We provide the requested information in an additional file.	Additional file 2
Conduct the following sensitivity analyses:<br>o Restrict analysis to patients presenting within GEM operating hours (“within-hours subset”) to test robustness<br>o Use doubly robust models (IPW + covariate adjustment)<br>o Explore alternative specifications (e.g., matching	With the help of the statistician, we have been able to conduct sensitivity analyses and these are now provided in the updated manuscript. We agree that these contribute to the robustness of our analyses. However, as mentioned in previous questions, we opted not to adjust for time of presentation but instead for prognostic factors that might differ due to presenting hours (such as disease severity).<br><br>As an alternative approach in our first sensitivity analysis, we recalculated our doubly robust approach with help from the statistician.<br>In addition, we now used g-computation as a third, alternative approach. This approach uses a conditional outcome regression model rather than a propensity score. In other words, the confounding is adjusted for directly rather than indirectly via design using the propensity score model for group assignment. Because of this	-Methods (analyses)<br>-Results<br>-Additional file 2

with calipers, entropy balancing).	difference in methodology, we argue it is a more meaningful alternative to our primary analysis using IPW, as these methods relies on different modelling and design assumptions. The results from this analysis were very similar to the primary analysis.	
Consider propensity-score matching rather than weighting, reporting matched-sample characteristics and effective sample size.	We thank the reviewer for this comment, please see the above reply regarding propensity score matching. We opted for g-computation instead, as that relies on different modelling and design assumptions, whereas matching would just use the same propensity score in a different way, and in so it does not help us to assess whether assumptions are met.	-
Report the distribution of propensity scores to confirm overlap.	Thank you for this request, we have now provided these.	Additional file 2 Results (descriptive characteristics)
Add the number of excluded unscreened patients.	Thank you for this request, we have now provided these in the text as well, these were originally only mentioned in the figure 2.	Results line 3
Include figure or table showing standardized mean differences before/after weighting.	Thank you for this request, we have now provided these. Please see also the previous question on this.	Additional file 2
